# Brain iron accumulation on MRI revealing aceruloplasminemia: a rare cause of simultaneous brain and systemic iron overload

**DOI:** 10.1259/bjrcr.20220035

**Published:** 2022-09-12

**Authors:** Firdaous Touarsa, Daoud Ali Mohamed, Behyamet Onka, Soufiane Rostoum, Najwa Ech-Cherif El Kettani, Meriem Fikri, Mohamed Jiddane

**Affiliations:** 1 Department of Neuroradiology, University Hospital Ibn Sina, Rabat, Morocco; 2 Department of Radiology, University Hospital Ibn Sina, Rabat, Morocco

## Abstract

Aceruloplasminemia is a rare autosomal recessive disorder of iron accumulation in the brain. It is one of the subtypes of Neurodegeneration with brain iron accumulation and is characterized by the uniform involvement of all basal ganglia, thalami, dentate nuclei, and cortex. Aceruloplasminemia is the only known iron overload disorder in which brain and systemic iron overload are combined. Here, we present a 53-year-old female who had progressive cognitive disorders with motor deficits. MRI showed extensive and abundant iron deposited in the brain and in the liver.

## Introduction

Neurodegeneration with brain iron accumulation (NBIA) is a group of diseases characterized by an abnormal accumulation of iron in the brain.^1,2^ Aceruloplasminemia (ACP) is one of the subtypes of NBIA and is characterized by the uniform involvement of all basal ganglia, thalami, dentate nuclei, cortex, and susbtantia nigra.^1,2^ ACP is a rare autosomal recessive disorder of iron accumulation in the brain caused by mutations in the ceruloplamin (CP) gene, which encodes CP, leading in turn to the absence or a strong reduction in CP activity.^3,4^ Here, we present a 53-year-old female who had progressive cognitive disorders with motor deficits. MRI showed iron deposited in the brain and in the liver.

## Clinical presentation

A 53-year-old female who presented with extremity tremor, bradykinesia, and plastic rigidity of the limbs. In her medical history, the patient was followed for mild chronic microcytic anemia since childhood, unlabeled, and refractory to iron supplementation. She was also hospitalized for anxiety, mood disorders, and unexplained bizarre behavior at age 45, according to the family (CT imaging not available, MRI was not done). Subsequently, the patient presented a progressive worsening of extremity tremor, bradykinesia, and plastic rigidity of the limbs. She had no other reported pathologies (no diabetes, no hypertension) or toxic habits.

## Investigations

**Figure 1.**
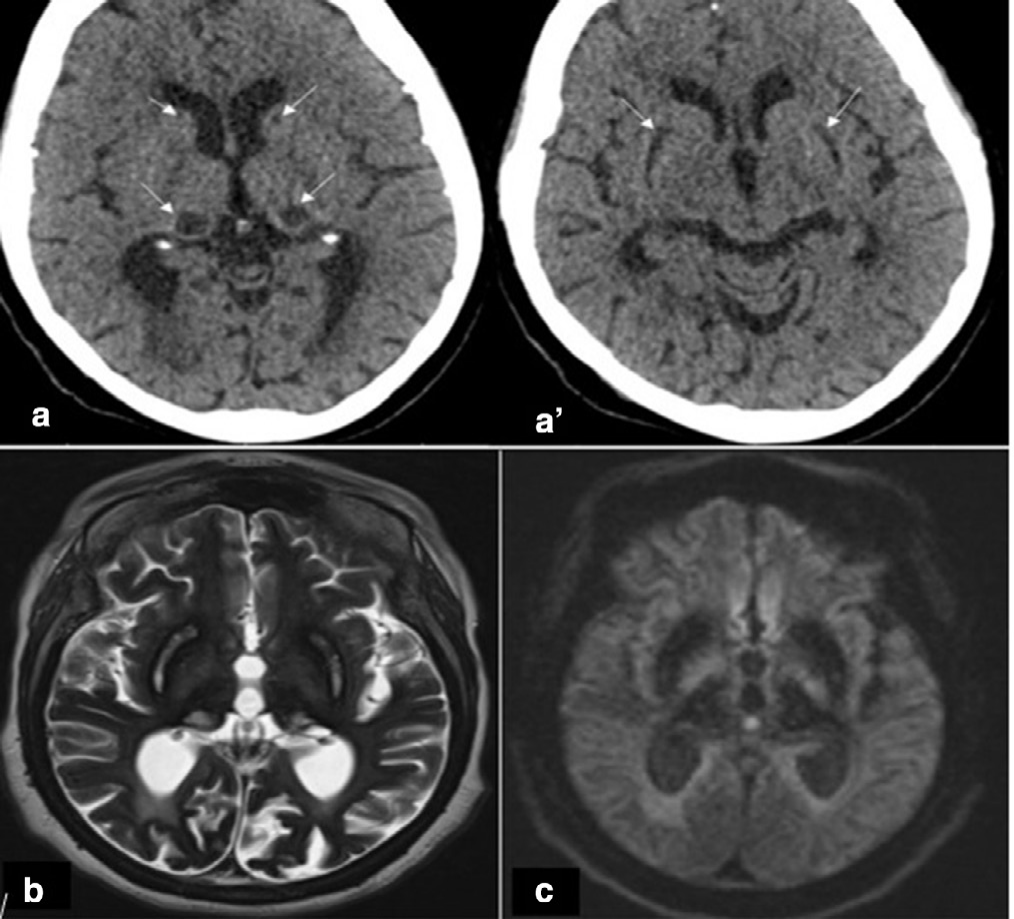
(A and A’) Axial CT scan showing hypodensity in the basal ganglia bilaterally (putamen, thalami and caudate). (B, C) Brain MRI (1,5 T): (B) Axial T2 showing hyperintense T2 signal in areas that were hypodense on CT surrounded by hypointense with also diffuse and bilateral hypointense cortices. (C) Axial diffusion showing hypo signal diffusion

**Figure 2.**
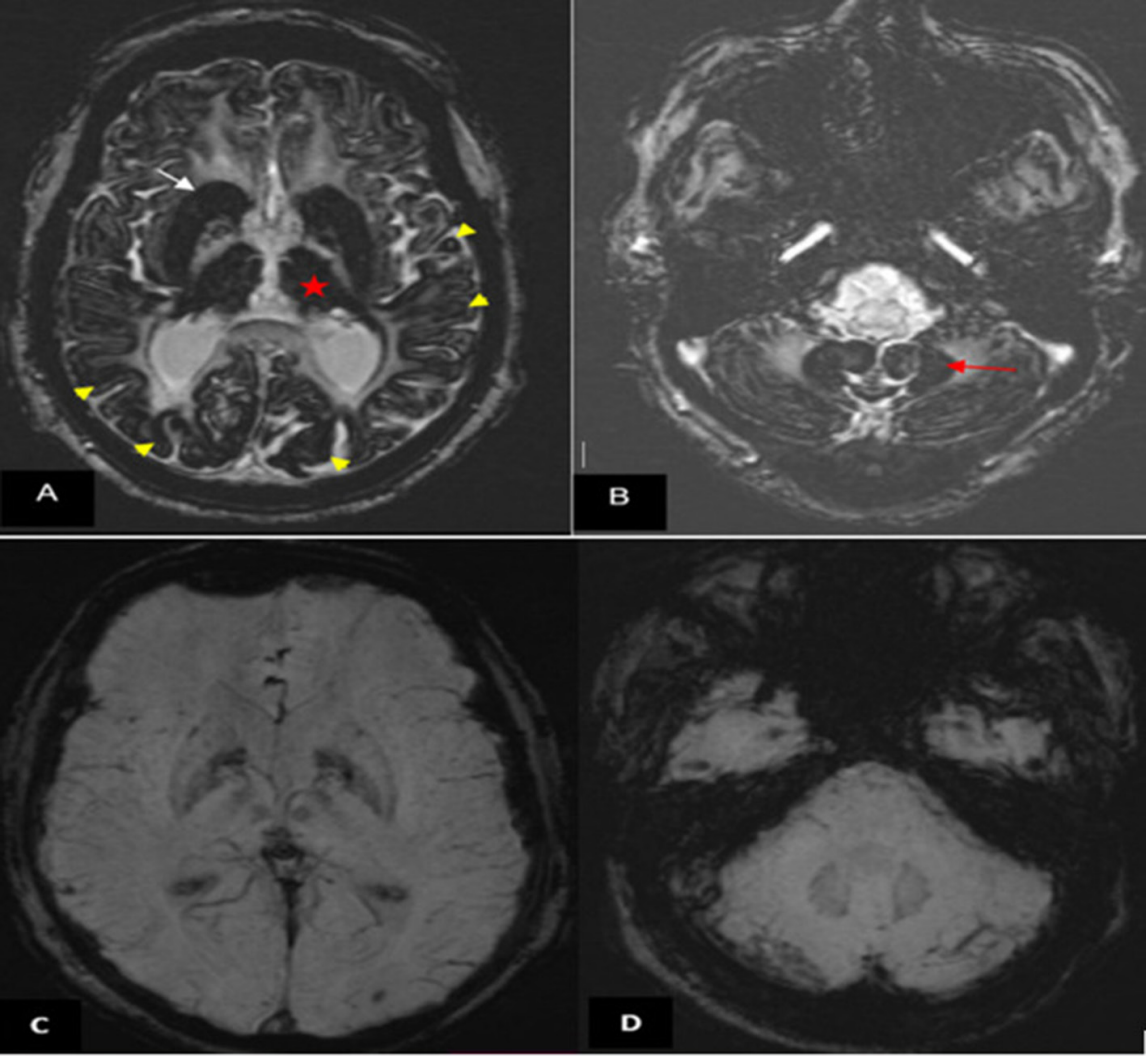
Brain MRI (1.5 T). (A, B) Axial SWI showing marked and extensive hypo intensity in the neo-striatum (white arrow), thalami (red star), cortex (yellow arrow head) (**A**) and dentate nuclei (red arrow (**B**)). (C, D) Normal SWI images in a matched patient followed for high blood pressure. SWI, susceptibility-weighted image.

The initial brain CT scan showed hypodense lesions in the basal ganglia bilaterally (putamen, thalami, and caudate). Given the patient’s medical history, with lesions centered on the basal ganglia on CT, an ischemic origin seemed unlikely. Therefore, a brain MRI was performed and showed hyperintensity on T2 (*T*_2_WI), hypointensity on T1 and diffusion-weighted image in the area that were hypodense on CT. These areas are surrounded by hypointesnity on *T*_2_WI and cortex bilaterally (Figure 1). The susceptibility-weighted image (SWI), showed extensive and symmetrical hypointenity involving the basal ganglia, thalamus, dentate nuclei, and cortex (Figure 2), suggesting NBIA, particularly the ACP.

The biological analysis (Table 1) showed microcytic anemia, low serum iron, high serum ferritin, and low transferrin saturation. Fasting glucose, liver enzymes, and other tests were normal. A ceruloplasmin assay was performed, which was found very low (0.09 g l^−1^), serum copper was relatively normal and 24 h urine copper was 22 μg/24 h, ruling out Wilson’s disease.

**Table 1.** Biological check-up in our case

Biological investigation	Results	Normal value
***Hematology***		
***Hemoglobin***	8 g dl^−1^	11, 5–15, 5 g dl^−1^
***MCV***	60,6 µm^3^	70–99 µm^3^
***Leukocyte***	4,3 x10^3/^ul	4,0-10,0 x10^3/^ul
***Platelet***	251 x10^3/^ul	150-400x10^3/^ul
***Iron studies***		
***Serum iron***	0, 27 mg l^−1^	0, 4–1, 45 mg l^−1^
***Transferrin saturation***	8,4%	15–50%
***Serum ferritin***	1537 ng ml^−1^	4–234 ng ml^−1^
***Biochemical***		
***Fasting glucose***	0,88 g l^−1^	0,7–1,1 g l^−1^
***AST***	24	15–45
***ALT***	18	10–40
***Total bilirubin***	7replace_with(7 mg/l^−1^)	2–12 mg l^−1^
***Serum creatine***	6,1replace_with(6,1 mg/l^−1^)	5,7–12,5 mg l^−1^
***Total protein***	63 g l^−1^	64–83 g l^−1^
***Serum CeruloplasminSerum copper***	0,09 g l^−1^ 1, 0 umol l^−1^ 0,06replace_with(0,06 mg/l^−1^)	0,22–0,5 g l^−1^ Over 6 month : 11,0–20,0 umol l^−1^ 0,7–1,27 mg l^−1^
***24 h urine copper***	23 ug l^−1^ 22 ug/24 h*	Normal < 20 ug l^−1^ Wilson disease suspicion > 100 ug/24 h

ALT, Alanine aminotransferase; AST, Aspartate aminotransferase; MCV, Mean corpuscular volume.

**Figure 3.**
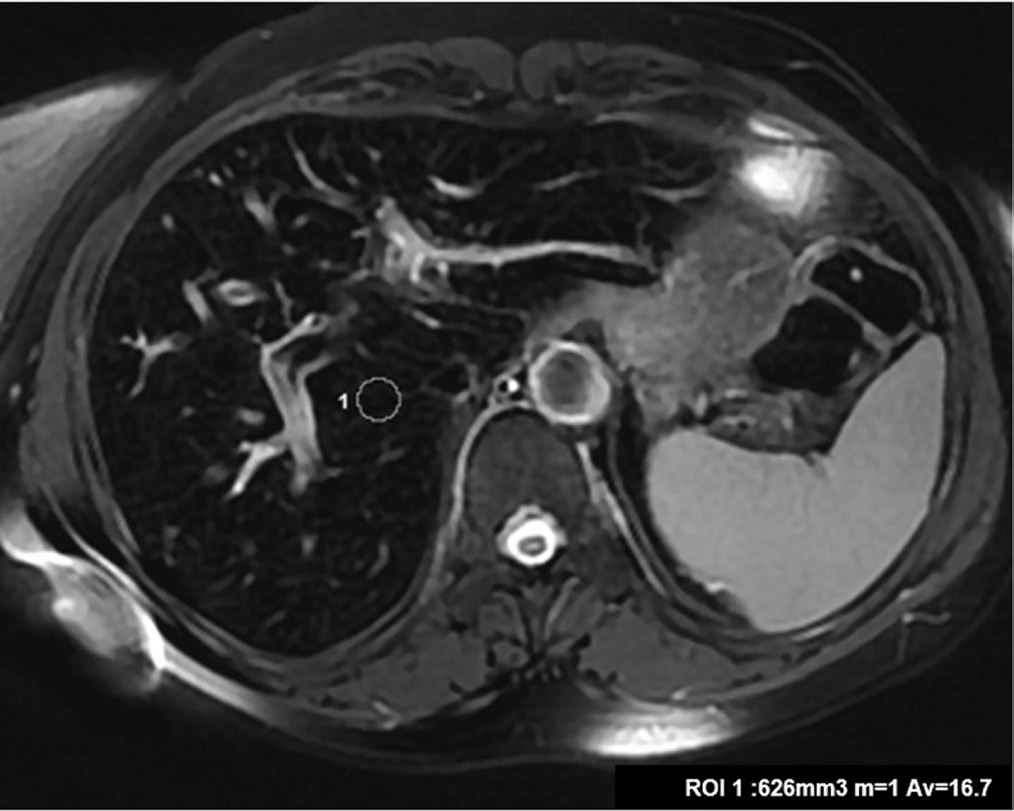
Liver MRI. Axial *T*_2_ weighted image showed marked hypointensity without hypointensity in the spleen.

Abdominal MRI revealed significant iron deposition in the liver without iron deposition in the spleen (Figure 3). On the basis of clinical and biological features, including simultaneous iron overload in the brain and liver on MRI, the diagnosis of ACP was retained. The patient was referred to a genetic counselor for discussion and testing, but was not yet performed.

## Discussion and differential diagnosis

ACP is a rare autosomal recessive disorder of iron accumulation in the brain caused by CP mutations that encode CP, which in turn leads to the absence or a strong reduction in CP activity.^3,4^ Ceruloplasmin is a copper transporter that transports more than 95% of copper, it also plays a role in the oxidation of ferrous iron (Fe 2+) into ferric iron (Fe 3+), thus avoiding the deposition of iron in organs (liver, pancreas, retina, and brain).^5^ ACP was first reported in 1987 in Japan by Miyajima, with prevalence estimated at 1/2,000,000 in Japan.^3^ It is the only one of the NBIAs in which brain and systemic iron overload is combined.^2,4^ In the brain, iron deposition is characterized by uniform involvement of all basal ganglia, thalami, dentate nuclei, and cortex, which is unique to this disease.^1,2,4^

**Figure 4.**
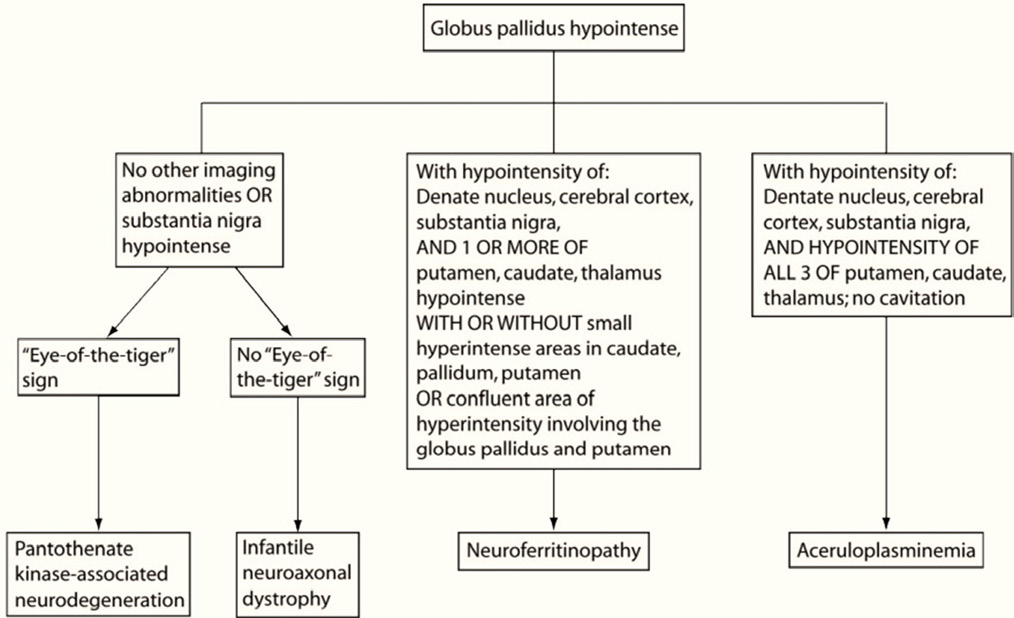
Simplified approach to microcytic anemia+high ferritin.^6^

Clinically, in the ACP, symptoms are variable and include, in frequency order, mild microcytic anemia, neurological impairment, diabetes in young people, retinopathy, and others.^4,6^ In ACP, mild microcytic anemia is the biological sign that appears in childhood, but is rarely diagnosed at this stage.^7,8^ Vila Cuenca et al proposed a simplified algorithm for the diagnosis of ACP.^6^ Microcytic anemia with elevated ferritin associated with low transferrin saturation is an indicator of early diagnosis of PCA especially when associated with neurological disorders. This would help prevent irreversible neurological complications. (Figure 4).^6^ Neurological disorders, diabetes, and retinopathy are the most common manifestations of most ACPs reported in the literature. Diabetes and neurological diseases are usually seen between the ages of 40 and 60.^4,7–9^_10

In the Japanese series, the triad of retinal degeneration, dementia, and diabetes is frequently cited, cerebellar disorders (dysarthria, trunk ataxia) and involuntary movements (dystonia, chorea, and tremor) in rare cases.^3^ However, in recent studies, Vroegindeweij et al reported cognitive impairment (apathy, memory loss), behavioral disturbances, and extrapyramidal signs observed most frequently in the Caucasian population.^9^ These non-specific neurological signs can often be underestimated.

**Figure 5.**
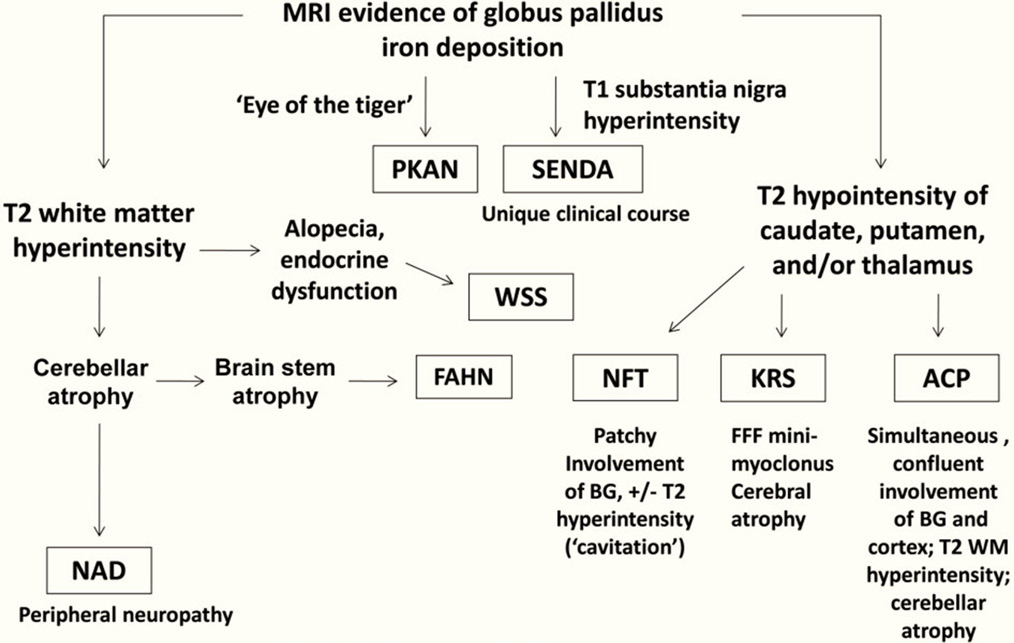
Neuroimaging features distinguishing subtypes of neurodegeneration.

**Figure 6.**
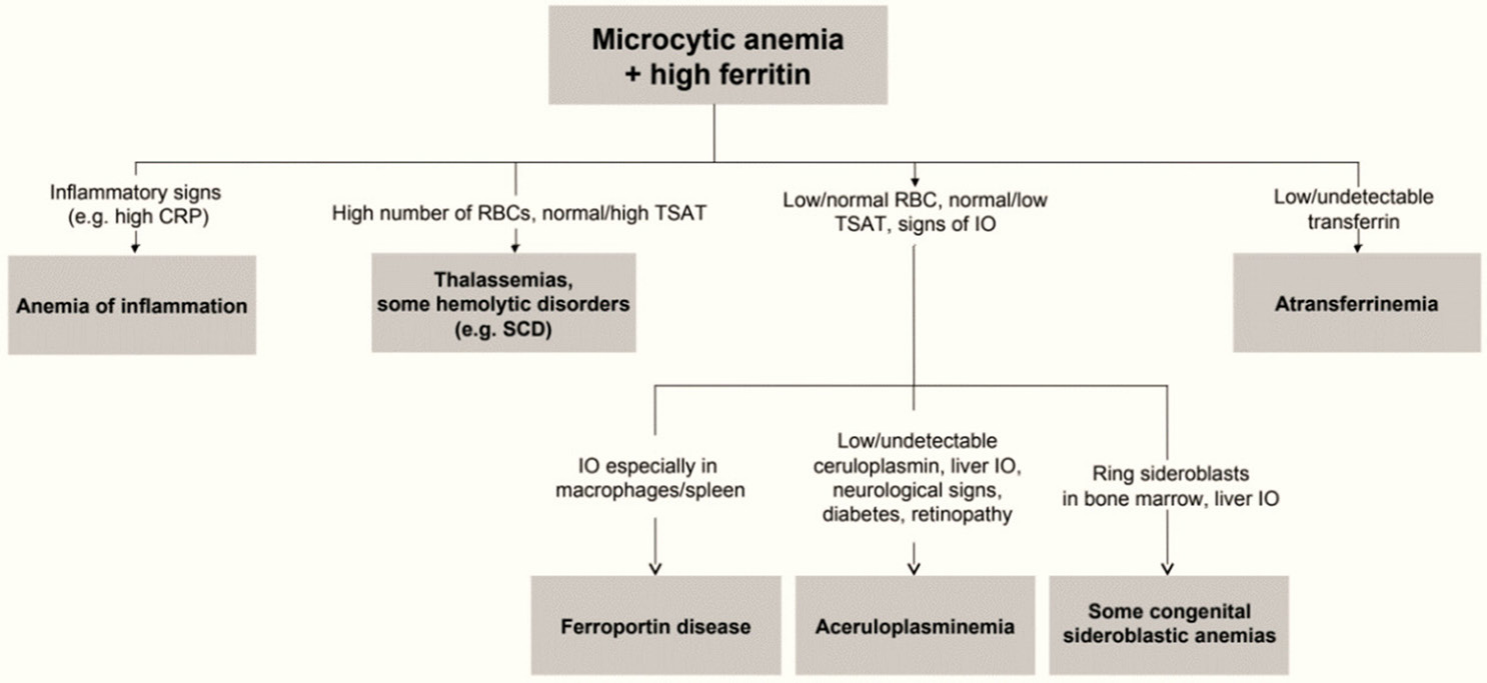
Clinical and neuroimaging-based algorithm for evaluating patients with suspected NBIA (Kruer el al [ref]. PKAN, phosphor-kinase associated neurodegeneration; SENDA, static encephalopathy of childhood with neurodegeneration in adulthood; WSS, Woodhouse-Sakati syndrome; NAD, neuroaxonal dystrophy; ACP, aceruloplasminemia; KRS, kufor-Rakeb syndrome; NFT, neuroferritinopathy; FAHN, fatty acid hydroxylase-associated neurodegeneration.

On brain imaging, all cases of NBIA show iron deposition, but differ in their clinical and neuroimaging features (Figures 5 and 6).^1,2,10^ To date, more than 10 disease entities related to NBIA disorders have been identified.^10,11^ PKAN is the most common form in which iron deposits are often restricted in the globus palludus characterized by the 'eye of the tiger' sign.^1,2,12^ In our case, the characteristic sign «eye of the tiger» was not observed. On MRI, eye of the tiger sign is the typical manifestation. *T*_2_ weighted sequences show hypointensity of the globus pallidus with a central hyperintensity, reflecting an excessive accumulation of iron with gliosis.^1,12^

Two neurodegenerative diseases with iron accumulation in the brain show great similarity: ACP and neuroferritionopathy (NFT) (Figures 5 and 6). In ACP, iron deposition is simultaneous and uniform in all basal ganglia, thalamus, dentate nuclei and cortex, substancia nigra, in the SWI sequence.^1,13^ In NFT, iron deposition can be multiple, but not simultaneously and uniform. Furthermore, in NFT there are cystic cavitations and areas of confluent hyperintensity T2 on MRI involving the global pallidus and putamen that are not seen in ACP.^1,14^ Biologically, in ACP, ferritinemia is high, in contrast to NFT where ferritinemia is normal or low.

Wilson’s disease was suspected on the basis of clinical features and low level of ceruloplasmin. But the appearance on the brain MRI was not compatible and the 24 h urine copper level was <100 μg/24 h, which ruled out this diagnosis.

ACP is the only known iron overload disorder in which cerebral and systemic iron overload are combined.^2,4^ Outside the brain, iron deposits are seen in the liver, but rarely result in clinically evident manifestations.^7,15^ In our case, the patient did not show signs of cirrhosis or hepatocellular insufficiency. Iron deposits have been reported sporadically in the pancreas, other endocrine glands, the heart, and others.^4,15^

The diagnosis of ACP is made by the absence or low level of ceruloplasmin, as its name suggests, as well as by clinical and biological signs (anemia+elevated ferritin with low transferrin saturation) and imaging signs (iron deposition).^4,10^ However, there are many other conditions with low levels of ceruloplasmin, including Wilson’s disease, Menkes disease, and others that remain differential diagnoses. Genetic study showing a mutation of the ceruloplasmin encoded gene located at the chromosomal locus 3q24-q25 confirms the diagnosis.^4,16^

## Learning points

- Aceruloplasminemia is a rare disease due to a deficiency or total absence of ceruloplasmin.
- Clinical manifestations are increasingly variable. Microcytic anemia with neurological manifestations (cognitive behavioral disorders with sometimes inconstant extrapyramidal signs) associated with high serum ferritin and low transferrin saturation should be investigated by brain MRI.
- Microcytic anemia with elevated serum ferritin and low transferrin saturation associated with neurological manifestations (cognitive, behavioral disorders with sometimes inconsistent extrapyramidal signs) can indicate aceruloplasminemia.
- Systemic iron deposits in the brain (basal ganglia, thalmi, dentate nuclei, putamen, and cortex) on brain MRI should suggest aceruloplasminemia and lead to the determination of ceruloplasmin.
